# Valproic acid and/or rapamycin preconditioning protects hair follicle stem cells from oxygen glucose serum deprivation-induced oxidative injury via activating Nrf2 pathway

**DOI:** 10.22099/mbrc.2024.49302.1922

**Published:** 2024

**Authors:** Fatemeh Keshavarzi, Mohammad Saied Salehi, Sareh Pandamooz, Razieh Zare, Mozhdeh Zamani, Zohreh Mostafavi-Pour, Pooneh Pooneh Mokarram

**Affiliations:** 1 Department of Biochemistry, School of Medicine, Shiraz University of Medical Sciences, Shiraz, Iran; 2 Clinical Neurology Research Center, Shiraz University of Medical Sciences, Shiraz, Iran; 3 Stem Cells Technology Research Center, Shiraz University of Medical Sciences, Shiraz, Iran; 4 Autophagy Research Center, Department of Biochemistry, School of Medicine, Shiraz University of Medical Sciences, Shiraz, Iran; 5 Maternal-Fetal Medicine Research Center, Shiraz University of Medical Sciences, Shiraz, Iran

**Keywords:** Ischemic stroke, Oxidative stress, Nrf2, Valproic acid, Rapamycin, Hair follicle stem cell

## Abstract

Among leading causes of the ischemic stroke pathogenesis, oxidative stress strongly declines rate of stem cell engraftment at the injury site, and disables stem cell-based therapy as a key treatment for ischemia stroke. To overcome this therapeutic limitation, preconditioning has been represented a possible approach to augment the adaptation and viability of stem cells to oxidative stress. Here, we illustrated protective impacts of valproic acid (VPA) and/or rapamycin (RAPA) preconditioning unto oxygen glucose and serum deprivation (OGSD)-stimulated cell damage in hair follicle-derived stem cells (HFSCs) and surveyed the plausible inducement mechanisms. OGSD, as an *in vitro *cell injury model, was established and HFSCs viability was observed using MTT assay after VPA, RAPA, and VPA-RAPA preconditioning under OGSD. ROS and MDA production was assessed to reflect oxidative stress. Real-time PCR and western blotting were employed to investigate Nrf2 expression. The activity of Nrf2-related antioxidant enzymes including NQO1, GPx and GSH level were examined. *VEGF* and *BDNF* mRNA expression levels were analyzed. Our results showed that VPA and/or RAPA preconditioning ameliorated OGSD-induced decline in HFSCs viability. In addition, they considerably prohibited ROS and MDA generation in the OGSD-treated HFSCs. Furthermore, VPA and/or RAPA preconditioning stimulated Nrf2 nuclear repositioning and NQO1 and GPx activity and GSH amount, as well as expression of paracrine factors *VEGF* and *BDNF* in OGSD-treated HFSCs. Thus, the protective effects afforded by VPA and/or RAPA preconditioning, which involved Nrf2-modulated oxidant stress and regulation of *VEGF* and *BDNF* expression, display a simple strategy to augment cell-transplantation efficiency for ischemic stroke.

## Introduction

As a significant resource for cell-based transplantation, hair follicle derived-stem cells (HFSCs) have recently been studied owning to their multi-directional differentiation ability and low immune reactivity [1]. HFSCs, reside in the bulge of hair follicle [2], remnant of embryonic neural crest [3], are associated ontologically with the central nervous system that exhibit high physiologic plasticity [4]. HFSCs can express different trophic cell factors which represent angiogenic, neuroprotective, and immunomodulatory potential in regenerative medicine [5]. HFSCs transplantation is extensively accepted for preclinical research in animal models of various ischemic conditions, such as cerebral ischemic stroke [6, 7]; however, satisfactory results were not yielded following transplantation, possibly due to cell dysfunction and massive cell death [8, 9]. The accelerated loss of stem cells post-transplantation might be related to oxidant stress, which is believed to be an important causes of stem cell death due to the extensive production of reactive oxygen species (ROS) following decreased blood flow (depletion of nutrients) and anoxia at the site of ischemic injury [10, 11]. Hence, attenuation of ROS production associated with oxidative stress during serum and glucose deprivation upon ischemia is necessity to improve HFSCs survival, thereby improving efficacy and therapeutic outcomes [12]. 

It has been shown that nuclear factor E2-related factor 2 (Nrf2) transcription factor, as the main regulator upstream of anti-oxidative response genes, is a significant means which protect cells against oxidative pathogenesis [13, 14]. Upon oxidative stress, the Kelch-like ECH-associated protein 1 (Keap-1) 's special cysteine residues are oxidized and Keap-1 loses its capability in Nrf2 ubiquitination and degradation, allowing Nrf2 entrance into the nucleus and bind to the antioxidant response elements (ARE) and initiate the antioxidative genes of interest expression, such as NAD(P) H:quinine oxidoreductase 1 (NQO1) and glutathione peroxidase (GPx) to remove excessive ROS [15, 16]. In this regard, various cell preconditioning tools have taken advantage of these Nrf2 properties to equip stem cells with such cytoprotective factor in order to prevent massive cell death after implantation [17, 18]. 

Earlier studies demonstrated that the Nrf2 expression level is influenced by the amount of histone acetylation and histone deacetylase (HDAC) enzymatic activity, as Nrf2 acetylated within p300/CBP enhances its ARE-binding capacity and modulates downstream transcription, indicating that Nrf2 acetylation might provide cellular protection [19, 20]. 

Giving the findings of Wang et al., valproic acid (VPA), well-known to prohibit HDACs, upregulates antioxidative responses, possibly due to enhanced Nrf2/ARE pathway activation, via direct HDAC3 inhibition post traumatic brain injury in rats [21]. VPA is a short chain fatty acid that possesses neurotrophic and neuroprotective properties in different neurological damage, such as brain ischemia, through chromatin hyperacetylation and induction of trophic/growth gene expression [22, 23]. Among trophic factors whose induction are governed by Nrf2, brain-derived neurotrophic factor (BDNF) and VEGF being pivotal regulators within post-stroke recovery, at least in part by inhibition of oxidant stress and subsequent neurotoxicity, maintaining a balance between anti-oxidation and oxidation mediators [24, 25]. In addition, both *VEGF* and *BDNF* are Nrf2-targeted genes; interestingly, it has proven that they can stimulate the Nrf2 signaling pathway [26-28]. Therefore, it seems that utilizing VPA preconditioning on HFSCs with antioxidative and neuroprotective properties, providing an ideal intervention for OGSD-induced oxidative stress. 

Rapamycin (RAPA) is currently known as an autophagy-specific inducer; however, under oxidative stress arising from the high production of endogenous ROS in ataxia [29] and pulmonary fibrosis [30], the protective impacts of RAPA are more probably associated with antioxidative virtues instead of autophagy stimulation. These RAPA-mediated protection versus oxidative conditions partly ascribed to an enhancement in the antioxidant genes transcription regulated through Nrf2 [29]. However, the underlying mechanism by which RAPA affects OGSD-induced oxidative stress remains unclear.

The main aim of this investigation was to determine the experimental basis for improving the effectiveness of HFSC-based therapy through small-molecule preconditioning. Therefore, we first employed the OGSD model to simulate *in vitro *ischemic disease microenvironment and then tested the hypothesis that if VPA and RAPA preconditioning protects HFSCs against OGSD-stimulated oxidant stress injury via inducing the Nrf2 signaling pathway and targeting *VEGF* and *BDNF* transcription.

## MATERIALS AND METHODS

**Materials and Reagents: **Valproic acid and rapamycin (50 mg/mL) were bought from RAHA Pharmaceutical Co. (Esfahan, Iran) and LC Laboratories (USA), respectively. Prior to each experiment, RAPA was prepared and diluted in dimethyl sulfoxide (DMSO;Sigma-Aldrich) and alpha-modified minimum essential medium (α-MEM, Bio Idea), respectively. Primary polyclonal rabbit antibodies such as anti-SOX10 (Proteintech, #10422-1-AP) and anti-nestin (Proteintech, #19483-1-AP) were provided by Proteintech Group, Inc., Germany, but anti-Nrf2 (Sc-20) was obtained from Santa Cruz Biotechnology, USA. A mouse primary antibody against glycerinaldehyde-3phosphate dehydrogenase (GAPDH, sc-47724) was obtained from Santa Cruz Biotechnology, USA. Anti-mouse IgG and anti-rabbit IgG peroxidase conjugated secondary antibodies were bought from Sigma-Aldrich (St. Louis, MO, USA). Also, a goat anti-rabbit IgG AlexaFluor488 secondary antibody was provided from Abcam (Cambridge, USA).

**The isolation and culture of HFSCs: **Based on a previously described method [31], hair follicles from the pubic hair skin punches (donated from a 22-year old brain-dead patient) were microdissected. Hair follicles were cut into small pieces and explanted on 4-well plates coated with collagen (1 mg/ml; Roche). The medium was composed of 10% fetal bovine serum, 10% chick embryonic extract, 1% penicillin/streptomycin and 1% L-glutamine in α-MEM. A few days post-plantation, the stem cells migrated from the follicles exhibited proliferation. HFSCs at passage 4-6 were employed. 

**Verification of HFSCs: **Immunostaining against SOX-10 (neural crest cell marker) and nestin (neural crest stem cell marker) was employed to confirm the identity of cultured HFSCs *in vitro *[4, 32, 33]. In brief, following fixation of migrated cells with paraformaldehyde 4%, normal goat serum was used to block them. Next, cells incubated with rabbit primary polyclonal antibodies anti-SOX10 (1:100) and anti-nestin (1:50), overnight. Immunostaining of each protein of interest was conducted individually, to prevent antibody cross-reactions. After washing and re-blocking, secondary antibody goat anti-rabbit IgG (AlexaFluor488, 1:1000) was used, and nuclei were counterstained with Hoechst 33342 (Sigma). Finally, immunofluoresc-ence images were photographed by a ZOE fluorescent cell imager.

**Oxygen glucose and serum deprivation (OGSD) model and preconditioning treatments: **In order to establish an OGSD experiment, HFSCs were covered with glucose and serum-free α-MEM and incubated in an anaerobic chamber with 5% CO_2_ and 95% N_2_ for 48 h at 37°C to simulate ischemic damage. HFSCs were cultured with 1mM VPA, 1µM RAPA, and 1mM VPA combined with 0.5µM RAPA for 72 h, followed by 48 h OGSD stimulation. All experiments on preconditioned and non-preconditioned HFSCs followed by OGSD and non-OGSD injury were performed to elucidate the underlying effects of VPA and/or RAPA preconditioning on ischemic stroke. 

**Cell survival test: **To determine stem cell survival MTT method was performed. Briefly, 2 × 10^4^ HFSCs were cultured in 96-well plates for a day and then pre-treated with VPA, RAPA, VPA-RAPA for 72 h, followed by 48 h OGSD. Afterward, each plate 'well was covered with MTT solution 0.5 mg/mL and placed for 4 h at 37°C. DMSO dissolved the formazan crystals in each well, and absorbance was measured using a microplate reader (Mikura Ltd.) at 570 nm. Cell survival percent was defined with regard to the control. MTT was repeated 3 times and each pre-treatment was tested in quadruplicate wells.

**Intracellular ROS level determination: **According to our previous publications, ROS generation in cells was investigated [13, 15]. Briefly, 2,7- dichlorofluorescein diacetate (DCFH-DA) was used as a fluorescent probe when entered into the cells and oxidized via H_2_O_2_ and O_2_ produced by the cells, converting to the fluorescent compound dichloride that can be measured by a fluorimeter. Here, 1×10^4^ HFSCs/well were cultured into the 96-wells plate. Following 72 h preconditioning followed by 48 h OGSD, plate' media was discarded and each plate' well was covered with 100 μl 10 μM DCFH-DA and incubated for 30 min at 37°C. After replacing the supernatant by 200 μl PBS, a fluorimeter (BMG LABTECH, Germany) was employed to determine fluorescence intensity of each well, using wavelengths 480 nm for the excitation and 530 nm for emission. 

**MDA level determination: **Based on colorimetric method, MDA level was analyzed. Briefly, in a boiling water bath, a mixture of 0.50 mL cell lysate' supernatant and 2 mL thiobarbituric acid (TBA) reagent (composing of TBA 0.37%, trichloroacetic acid 15%, and HCl 0.25 mol/L) was heated for 15 minutes. After cooling the mixture, it was centrifuged at 8000 × g for 15 minutes at 4 °C. The pink supernatant absorbance was assessed at 532 nm. Finally, for calculating the MDA concentration results, tetraethoxypropane (TEP) was employed as the standard and reported as nmol/mg protein.

**NQO1 activity determination: **The NQO1 activity was measured spectrophotometrically, in accordance with our previous publications [13, 15]. Here, HFSCs before being exposed to OGSD cultured and pre-treated with VPA, RAPA, VPA-RAPA for 72 h. After OGSD for 48 h, 2 ml 250 mM sucrose and 25 mM Tris-HCl buffer (pH 7.4) were added to the stem cells and the cells were lysed by sonicator (4 times, each time 10 s on ice). Next, lysate of stem cells was centrifuged at 10,000 g for 30 s at 4°C and enzyme activity was measured using supernatant. When an electron donor, NADH was oxidized, an electron acceptor, dichloroindophenol (DCPIP), was reduced by NQO1 enzyme (presented in the supernatant) and lost its blue color at 600 nm over 2 min, which switching color was associated to the enzyme activity (DCPIP reduction depend on NADH). As a terminator and inhibitor of the reaction, dicoumarol was employed. Finally, activity of NQO1 was expressed as nM/min/mg protein.

**GSH level determination: **GSH level was measured using the 5,5dithiobis(2-trobenzoic acid) (DTNB), in accordance with the Ellman's method. 1 mM GSH solution was used to construct a standard curve. Here, after OGSD or non-OGSD injury, preconditioned or un-preconditioned stem cells were lysed and their clear supernatant was employed for GSH amount measurement. Regarding, 0.5 mL 0.001 M DTNB was added to a mixture of 2.3 mL, 0.2 M PBS (potassium phosphate buffer, pH 7.6) and cell lysate supernatant (0.2 ml), and then absorbance of solution was read after 5 minutes at 412 nm.

**GPx activity determination: **Based on the previously described procedure by Fecondo and Augusteyn, GPx activity was determined through screening the continuous re-generation of GSH (reduced glutathione) from GSSG (oxidized glutathione), in the disodium salt presence, reduced NADPH (nicotinamide adenine dinucleotide-phosphate), and GR (glutathione reductase). Here, after preconditioning of HFSCs followed by OGSD or non-OGSD, stem cells were lysed and clear supernatant was employed to measure GPx activity, reported as μmol of oxidized NADPH/min/mg cell protein by a molar extinction coefficient of 6.22 × 10^6^ M^1^ cm^1^ for NADPH. Finally, mU/mg cell protein defined one unit of GPx.

**Real-time PCR: **Whole RNA of preconditioned and un-preconditioned HFSCs followed with or without OGSD, was extracted and then converted to cDNA using the Tripure reagent and the cDNA Synthesis Kit (Fermentas; Thermo Fisher Scientific, Inc.), according to the supplier's instruction. Quantitative Real time-PCR was done in triplicate using SYBR-Green PCR Master-Mix and a Quant-Studio Real-time-PCR-System with the specific primers (Table 1). The relative expression of *Nrf2* and paracrine factors *VEGF* and *BDNF* were calculated by the 2^−^^△△^^Ct^ formula. The interest genes were normalized using *GAPDH* as an internal control. A 7500 Software v 2.0.01 was employed to analyze the results. 

**Table 1:** Gene specific primers

**Target Gene**	**Forward primer**	**Reverse primer**
*GAPDH*	CTGAAGTAGTGATGATAACAA	AAGAGATGATGTCCAACC
*Nrf2*	ACA CGG TCC ACA GCT CAT C	TGT CAA TCA AAT CCA TGT CCT G
*VEGF*	ACTTGAGTTGGGAGGAGGATGTC	GGATGGGTTTGTCGTGTTTCTGG
*BDNF*	CGATTAGGTGGCTTCATAGGAGAC	CAGAACAGAACAGAACAGAACAGG

**Western blot analysis: **All steps of western blotting processes were performed in accordance with our previous investigation [15]. In brief, stem cells preconditioned with VPA, RAPA, and VPA-RAPA for 72 h and then exposed to OGSD stress. Afterward, the nuclear and cytoplasmic proteins of preconditioned and un-preconditioned cells with and without OGSD were prepared by NE-PER Cytoplasmic and Nuclear Extraction Reagents Kit (Thermo Scientific, USA) and quantified by the BCA Protein Assay Kit (Thermo Scientific, USA). 30 μg of extracted proteins were electrophoresed on 15% SDS-PAGE gel and then blotted onto 0.2 μm nitrocellulose membranes, which blocking in 5% free skimmed milk for 2 h. After membrane exposing with primary antibodies anti-Nrf2 (1:1000) and anti-GAPDH (as internal control, 1:1000) for a night at 4°C, they were covered with corresponding HRP conjugated secondary antibody for 3 h at 25°C. Ultimately, protein bands on the treated membranes by enhanced chemiluminescence reagent (Abcam, USA) were visualised using Chemi-DocTM-MP imaging equipment (Bio-Rad, CA) and semi-quantified by Image-Lab software which normalized to GAPDH. 

**Statistical analysis: **Three independent assays' mean ± SD data were examined using Graph-Pad Prism software (Version 6.01, San Diego, CA) with one-way ANOVA and the Tukey post-hoc test. P<0.05 was regarded as statistically significant.

## Results

**Figure 1:**
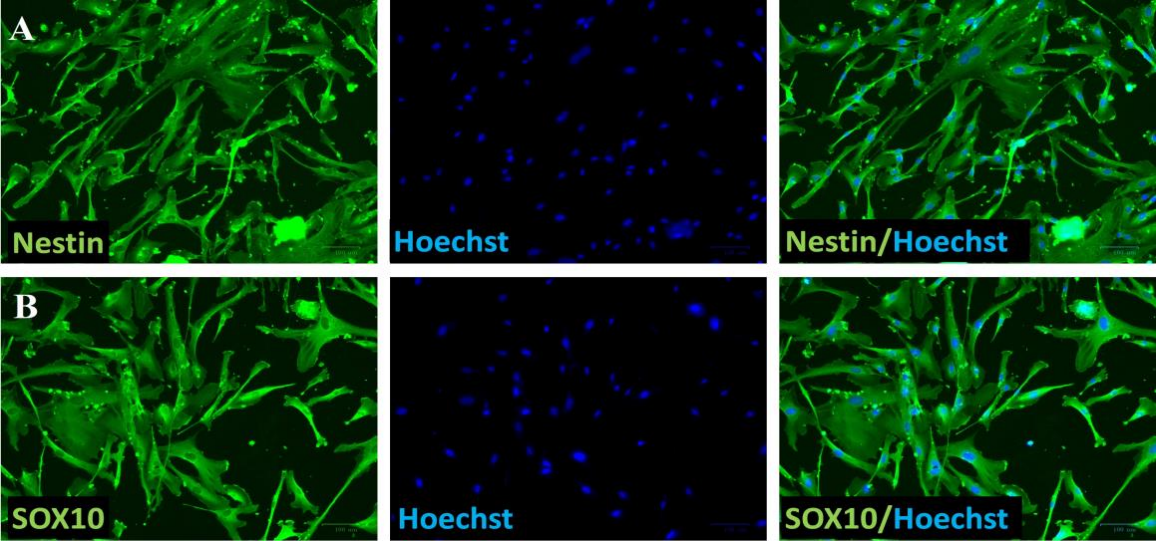
The characterization of human hair follicle stem cells. Hair follicles were microdissected from pubic hair skin punches and explanted onto cell culture plates. The immunostaining showed that migrating cells displayed significant levels of nestin (**A**) and SOX10 (**B**). Hoechst counterstained cell nuclei. Images serve as illustrations of three evaluations that were conducted for each immunostaining (n=3).

Microdissected hair follicles were explanted onto cell culture plates from pubic hair skin punches. Cells were found around the follicles 3-5 days after explantation and proliferated over time. Immunostaining demonstrated that migrating cells displayed significant level of nestin as neural crest stem-cell marker (Fig. 1A), and SOX10 as neural crest cell marker (Fig. 1B).

**Figure 2:**
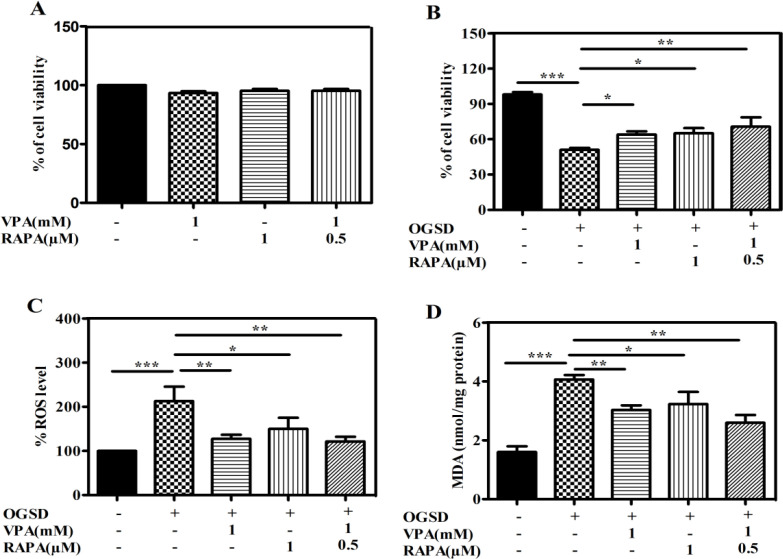
Protective effect of VPA, RAPA, and VPA-RAPA preconditioning on OGSD-induced HFSCs injury. **(A)** HFSCs were preconditioned with 1mM VPA, 1µM RAPA, and 1mM VPA plus 0.5µM RAPA for 72 h. Cell viability was measured using MTT assay. **(B)** HFSCs were preconditioned with 1mM VPA, 1µM RAPA, and 1mM VPA plus 0.5µM RAPA for 72 h, followed by OGSD stimulation. Cell viability was measured using MTT assay. Values are reported as mean±SD. Anti-oxidative effect of VPA and/or RAPA preconditioning on OGSD-induced HFSCs. HFSCs were preconditioned with 1mM VPA, 1µM RAPA, and 1mM VPA plus 0.5µM RAPA for 72 h, followed by OGSD stimulation. **(C)** Effect of VPA and/or RAPA preconditioning on ROS generation. **(D)** Effect of VPA and/or RAPA preconditioning on MDA production. Data are expressed as mean±SD. (n=3, p<0.001***, p<0.01**, p<0.05*).

In our previous study we examined cell viability of VPA and RAPA on HFSCs by MTT assay and then concentration of 1mM VPA, 1µM RAPA, and 1mM VPA combined by 0.5µM RAPA were chosen as suitable doses for HFSCs preconditioning. As shown in Figure 2A, these concentrations of VPA and RAPA showed no significant change on HFSCs viability. So, in the current study, in order to survey the cytoprotection of VPA, RAPA, VPA-RAPA, we used HFSCs with or without preconditioning for 72 h followed by 48 h OGSD or non-OGSD injury. As illustrated in Figure 2B, HFSCs survival was 97.0% in the non-OGSD group and remarkably reduced to 51.0% in the OGSD group (p<0.001). However, preconditioning of VPA and RAPA were able to considerably enhance the cell survival of OGSD-received HFSCs to 64.0% and 66%, respectively (p<0.05). Surprisingly, HFSCs preconditioning by VPA-RAPA bringing cell viability close to 70% (p<0.01). 

Earlier investigations have proven that OGSD is cable to stimulate oxidative stress [34]. Here, in order to survey the VPA and RAPA preconditioning' effects on OGSD-stimulated oxidative stress, the MDA and ROS generation (as oxidant stress indicators) were measured. As shown in Figure 2B, OGSD-received cells generated considerably more levels of ROS in comparison to the non-OGSD cells (p<0.001), however, VPA, RAPA, VPA-RAPA pre-treatment markedly decreased the ROS amounts in the preconditioned cells in comparison to the OGSD cells (p<0.01, p<0.05, p<0.01, respectively). Similarly, as shown in Figure 2C, the amounts of MDA was more in the OGSD-received cells than the non-OGSD cells (p<0.001), but this effect significantly was reversed by VPA (p<0.01), RAPA (p>0.05), VPA-RAPA (p<0.01) preconditioning. It is noteworthy that compared to either VPA or RAPA alone, VPA combined with RAPA pretreatment showed better efficiency in reducing both ROS and MDA.

Nrf2 signaling pathway is a main pathway that is involved in oxidant stress [35]. Hence, *Nrf2* mRNA expression level was measured in HFSCs with or without preconditioning after exposing to non-OGSD or OGSD damage. Also, western blotting was employed to determine Nrf2 protein amounts in both nuclear and cytoplasm extracts. 

**Figure 3:**
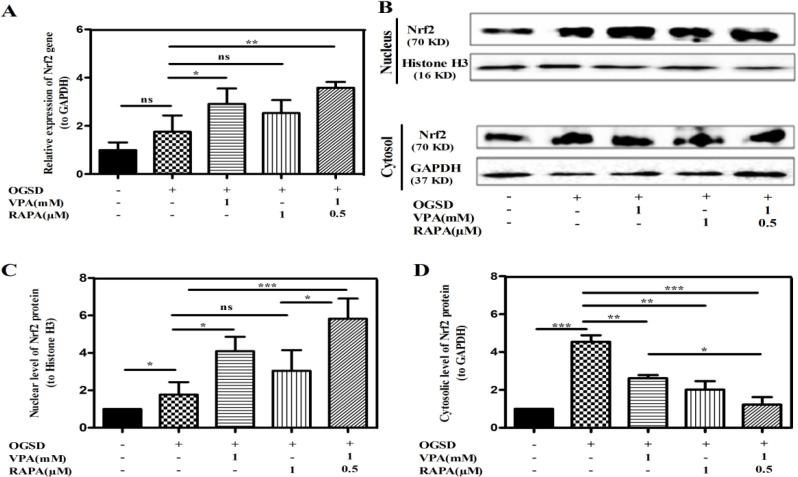
VPA and RAPA preconditioning either alone or in combination enhanced the expression and nuclear translocation of Nrf2 in HFSCs 48 h after OGSD injury. HFSCs were incubated with 1mM VPA, 1 µM RAPA, and 1mM VPA plus 0.5µM RAPA for 72 h prior to OGSD stimulation. **(A)** Relative mRNA expression level of *Nrf2* was measured by Real-time PCR. *GAPDH* served as the loading control. **(B)** Representative western blot image showing the Nrf2 protein expression in nucleus and cytosol of HFSCs after OGSD. **(C)** Graph of quantification analysis of the protein expression of nuclear Nrf2/histone H3. **(D)** Graph of quantification analysis of the protein expression of cytosolic Nrf2/GAPDH. Values are shown as the mean ± SD (n=3). (*P<0.05; **P<0.01; ***P<0.001)

Acquiring results from Real time-PCR shown in Figure 3A indicated that compared to the non-OGSD group, an enhancement in *Nrf2* mRNA level was noticed in OGSD group. Also, pre-treatment with VPA, RAPA, and VPA-RAPA significantly induced this enhanced *Nrf2* transcription as compared with OGSD-treated HFSCs (p<0.05, p>0.05, p<0.01). 

Furthermore, western blotting data depicted in Figure 3B-C showed that the OGSD-received cells remarkably noticed higher Nrf2 protein in the nucleus than the non-OGSD cells (p<0.05), indicating the Nrf2 nuclear transference post OGSD damage. Indeed, Nrf2 was located mainly in the cytoplasm of OGSD cells (p<0.001, Fig. 3D), however, nuclear transposition after 72 h HFSCs pre-treatment with VPA, RAPA and VPA-RAPA was evident and Nrf2 switched to being primarily localized at the nuclei (p<0.05, p>0.05, p<0.001 vs. OGSD cells, Fig. 3C). Interestingly, co-pretreatment with VPA and RAPA more potentially facilitated Nrf2 transference to the nucleus than each one alone (p<0.05, Fig. 3C). 

**Figure 4:**
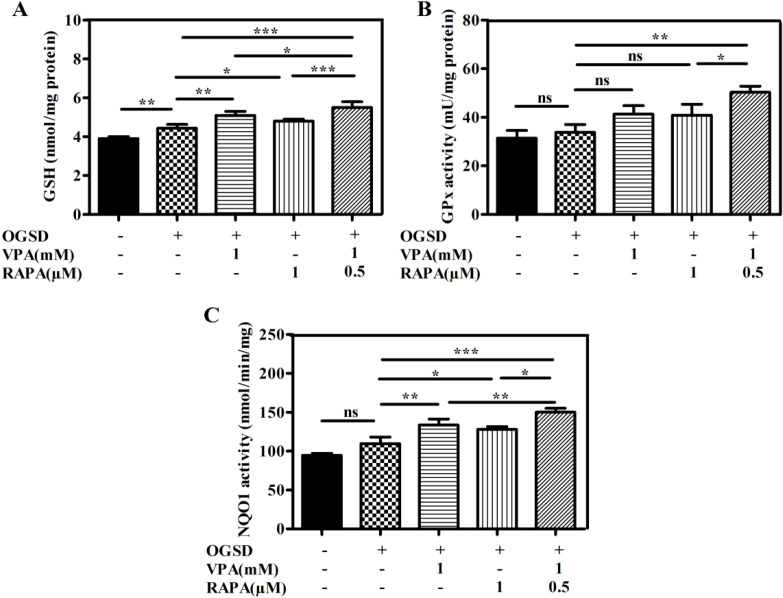
VPA and RAPA preconditioning either alone or in combination enhanced the activity of Nrf2 downstream protein in OGSD-induced HFSCs. HFSCs were incubated with pre-treatment of 1mM VPA, 1 µM RAPA, and 1mM VPA plus 0.5µM RAPA for 72 h prior to OGSD stimulation and then **(A)** GSH level and **(B)** GPx activity and **(C)** NQO1 activity were measured. Data are expressed as mean ± SD (n = 3). (*P < 0.05; **P < 0.01; ***P < 0.001).

Next, we investigated if VPA and/or RAPA preconditioning on HFSCs affected the Nrf2' downstream enzyme activity including NQO1, GPx, and GSH. Figure 4A shows that OGSD injury increased the GSH level, a significant non-enzymatic defense, in the OGSD cells in comparison to the non-OGSD cells (p<0.01), although, VPA, RAPA, VPA-RAPA preconditioning markedly increased the enhanced GSH amounts in the preconditioned cells compared to the unpreconditioned-OGSD cells (p<0.01, p<0.05, p<0.001, respectively). Notably, co-pretreatment with VPA and RAPA more significantly increased GSH compared to each VPA (p<0.05) or RAPA (p<0.001) alone. Also, Figure 4B indicates the level of GPx was not significant increased after HFSCs being exposed to OGSD (p>0.05). But, co-preconditioning with VPA-RAPA increased considerably the enhanced GPx in OGSD-treated HFSCs (p<0.01). Similarly, Figure 4C shows that OGSD induced the activity of NQO1. However, the increased activity of this enzyme was enhanced by VPA (p<0.01), RAPA (p<0.05), and VPA-RAPA (p<0.001) preconditioning. Furthermore, most prominent enhancement of NQO1 activity was observed in the VPA (p<0.01) and RAPA (p<0.05) coprimed HFSCs compared to each one alone.

**Figure 5:**
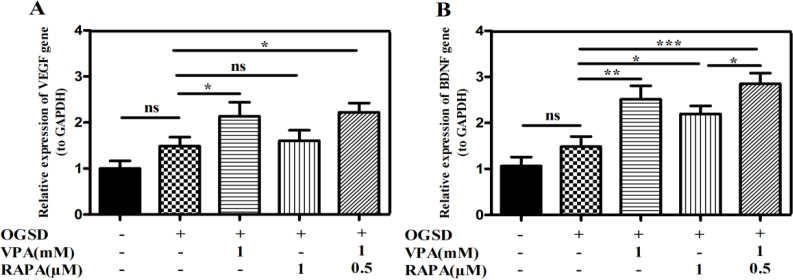
VPA and/or RAPA preconditioning up-regulated the expression of paracrine factors in vitro. HFSCs were incubated with pre-treatment of 1mM VPA, 1 µM RAPA, and 1mM VPA plus 0.5µM RAPA for 72 h prior to OGSD stimulation and then mRNA expression level of **(A)** *VEGF* and **(B)** *BDNF* was assessed using Real-time PCR. *GAPDH* served as the loading control. The data is reported as the mean ± SD of three independent tests. (*P<0.05; **P<0.01; ***P<0.001).

Since expression of paracrine factors could be regulated through Nrf2 in other types of cells [24, 36], we investigated whether VPA and/or RAPA preconditioning modified their expression in HFSCs. Significantly further gene transcription of *VEGF* and *BDNF* was determined in the preconditioned-HFSCs after being exposed to OGSD compared with the non-preconditioned HFSCs (Figs. 5A-B). As shown in Figure 5A, VPA and RAPA pretreatment increased the transcript level of *VEGF* by 2.12 fold (p<0.05) and 1.6 fold (p>0.05), respectively in compared to non-pretreatment cells. The VPA-RAPA co-pretreatment group also induced a significant increase in the *VEGF* after 72 h (2.22 fold P<0.05). Also, as illustrated in Figure 5B, compared to non-pretreated HFSCs, VPA and RAPA, and VPA-RAPA preconditioning on HFSCs induced *BDNF* expression level by 2.5 fold (p<0.01), 1.2 fold (p<0.05), and 2.7 fold (p<0.001), respectively. This up-regulation was prominent increased by both VPA and RAPA precondi-tioning compared with each ones alone (p<0.05).

## Discussion

In our study, when HFSCs were subjected to OGSD, we observed that VPA and/or RAPA preconditioning resulted in an increase in cell survival significantly, consistent to our previous study. This preconditioning' cell protection associated to an oxidative stress reduction, recognized to happen during OGSD injury. To our knowledge, this is the first evidence that VPA and/or RAPA preconditioning stimulated Nrf2 nuclear transference and promoted the amounts of antioxidants, as well as trophic factors expression such as *VEGF* and *BDNF*. 

HFSCs also called epidermal neural crest stem cells (EPINCSCs) support the repair of spinal cord injury [37] peripheral nerve defect [38] and cerebral ischemic stroke in animal models [7]. Up to now, different cell preconditioning tools have been employed to optimize the functionality of HFSCs *in vitro *[1, 39]. Given the finding of our previous study, VPA and/or RAPA preconditioning associated with HFSCs having a remarkably better survival capacity and an increased ability to respond to OGD injury; these properties partially ascribed to autophagy induction. However, others cellular mechanism underpinning the adaptation and viability of HFSCs through VPA and RAPA-preconditioning during OGD-induced injury are unknown.

Since oxidative stress is one of the main reasons why stem cells die following transplantation, several studies have already investigated the beneficial impact of stem cell preconditioning with antioxidant stress substance [24, 40]; thus, in this study, we evaluated the role of oxidant stress in OGSD-stimulated injury and the cytoprotective impacts of VPA and RAPA preconditioning on HFSCs. 

Indeed oxidative stress during the OGSD process resulted from overwhelming of antioxidant systems versus excessive ROS production [41]. In order to explore oxidative stress following OGSD and verifying the possible anti-oxidant effects of VPA and RAPA, the generation of oxidant stress markers mainly the ones ROS and MDA were measured, both in preconditioned and non preconditioned stem cells. Our results demonstrated that precondition-ing with VPA or RAPA alone attenuated OGSD-induced increases in ROS and MDA in HFSCs-treated OGSD. Notably, these results additively changed in HFSCs co-priming alongside both VPA and RAPA. Our finding are in line with earlier studies that demonstrated that RAPA pretreatment protects AD-MSCs against cisplatin-induced renotoxicity, confirming by a substantial decrease in the level of MDA and ROS [42] and also priming with VPA increases BM-MSC's capacity to control the steady-state amounts of ROS and protecting against H2O2-induced oxidative damage, providing an excellent chance to survive engraftment [43]. Intracellular antioxidant pathways which regulated by VPA and RAPA have been investigated, although with un-precise outcomes.

Growing body of evidences all indicated that Nrf2 is pivotal in terms of defense against oxidant stress and cell death during stressed ischemic context [44]. Zhang et al. demonstrated that Nrf2 knockout mice considerably enhanced the ischemic stroke related to oxidative pathology [45]. According to Chen et al.’s study, VPA-mediated antioxidant responses following traumatic brain injury (TBI) are possibly associated to enhanced activity of Nrf2/ARE pathway, via inhibiting HDAC3, thereby leading to neuroprotection [21]. Meanwhile, Tai et al. explored that the RAPA' protective impacts in paraquat (PQ)-induced pulmonary fibrosis are likely associated with the activation of the Nrf2 and its transference to the nuclei, where in turn increasing the antioxidative downstream genes transcription [30].

Regarding to our study, we also showed that level of Nrf2 expression along with its downstream antioxidant protein activity (i.e., NQO1, GPx, and GSH) elevated in stem cells exposed to OGSD injury compared with the non OGSD-exposed may be due to cell’s own capability to resist oxidative stress, nonetheless these evidences were remarkably up-regulated when HFSCs pretreated with VAPA and RAPA alone or in combination. Additionally, based on the outcomes of the western blotting, we discovered VPA and RAPA preconditioning strongly facilitated Nrf2 translocation to the nucleus of HFSCs which received OGSD injury. It is noteworthy that co-priming with RAPA and VPA robustly increased this efficiency. Collectively, since active Nrf2 is a key coordinator for the stem cells under OGSD damage, we think that Nrf2-initiated antioxidants is an important event in HFSCs for governing cell homeostasis upon oxidant stressed condition. Importantly, no study investigating the Nrf2 antioxidative' effect induced by combined VPA-RAPA preconditioning has been conducted until now.

Naïve HFSCs express different paracrine factors mainly the individual ones *VEGF* and *BDNF* being among them [46], whose neurotrophic privilege extensively demonstrated in the devastative conditions of ischemic stroke animal models [47, 48]. Although, stem cell based implantation was designed to replace the degenerated neurons in neurodegenerative disorders, newly investigations indicated that the post-engraftment recovery is mainly ascribed to the secretion and replenishment of numerous neurotrophins [49, 50]. Hence, the ongoing studies have been established unto the augmentation of neurotrophins in stem cells before engraftment to gain an optimized recovery [51]. Surprisingly our finding indicated that VPA and/or RAPA preconditioning up-regulated considerably the baseline expression amounts of both *BDNF* and *VEGF* in HFSCs. Although we recently showed enhanced *VEGF* and *BDNF* expression levels in rat derived-HFSCs following VPA treatment [52], here, we indicated that the both *VEGF* and *BDNF* mRNA expression were more robustly upregulated if the VPA was applied with RAPA on HFSCs. In light of previous studies' results indicating that genetically modified stem cells that over-expressing special neurotrophins is neuroprotective [53], we suggested that engrafment of VPA and RAPA preconditioned-HFSCs could be more appealing due to stably expressing individual paracrine factors.

While earlier studies claimed that Nrf2 is contributed to the stimulation of neurotrophins, it is imperative for us to conduct further research on HFSCs to determine the transcriptional regulation of paracrine factors *VEGF* and *BDNF* in the Nrf2-dependent manner upon VPA and/or RAPA preconditioning. 

## Conflict of Interest:

The authors declare no conflict of interest.

## Authors’ Contribution:

FK did all experiments, statistical analysis, figure preparation, and prepared the first draft of the manuscript. MSS and SP isolated and cultured the stem cells, and advised us on the OGSD process. MZ and RZ were contributors in writing the manuscript. PM and ZM supervised FK, designed the project, supervised the project, did a final proof of the manuscript, and provided financial support for the project. All authors have read and agreed to the published version of the manuscript.
